# Cardiac MRI is safe in patients with pacemakers and defibrillators

**DOI:** 10.1186/1532-429X-14-S1-O102

**Published:** 2012-02-01

**Authors:** Alex Baher, Santhisri Kodali, Jiaqiong Xu, William A Zoghbi, Miguel Quinones, Miguel Valderrábano, Dipan J Shah

**Affiliations:** 1 Internal Medicine, The Methodist Hospital, Houston, TX, USA; 2 Methodist DeBakey Heart & Vascular Center, The Methodist Hospital, Houston, TX, USA

## Background

MRI has generally been contraindicated in patients (pts) with pacemakers (PM) or defibrillators (ICD) due to concern of a variety of potential complications. Several recent series have suggested potential safety of MRI in pts with cardiac devices, but most excluded cardiac or thoracic imaging. Additionally there is very limited data on safety of CMR in pts with devices, especially in pts with ICD.

## Methods

Pts with devices underwent CMR on a 1.5-T Siemens Avanto scanner utilizing a pre-specified protocol that involved device interrogation and programming prior to, and immediately after CMR to assess lead impedance, battery voltage, and pacing capture/sensing thresholds. Devices in PM dependent pts (intrinsic HR < 60 bpm) were reprogrammed to asynchronous pacing mode, whereas devices in non-PM dependent pts were reprogrammed to pacing function deactivated. All underwent rhythm, pulse oximetry, and visual monitoring during CMR. All pts were scheduled for follow up device interrogation to evaluate for later changes in device parameters. For hospitalized pts troponin levels were measured prior to and 8 hours after CMR.

## Results

Seventy nine CMR exams were performed in 64 pts [26 men, age 64.7±14.2 years, 34 with PM, and 30 with ICD]. Of these, 47 exams [24 with PM, and 23 with ICD] were on inpatients who had troponin levels drawn before and after CMR. All pts underwent CMR without complications, specifically none complained of heating, and there were no malignant arrhythmias or device malfunctions noted. Follow up device interrogation was performed after 68 (86%) of the exams at 13.2 ± 20.8 days after CMR. No device complications were noted during the follow up period. Table 1 and Table 2 summarize the changes in device parameters. We detected no significant rise in troponin levels with CMR (0.046 ± 0.200 and 0.037 ± 0.132, p = 0.596 for pts with PM; and value 0.099 ± 0.263 and 0.108 ± 0.247, p = 0.434 for pts with ICD).

**Figure 1:**
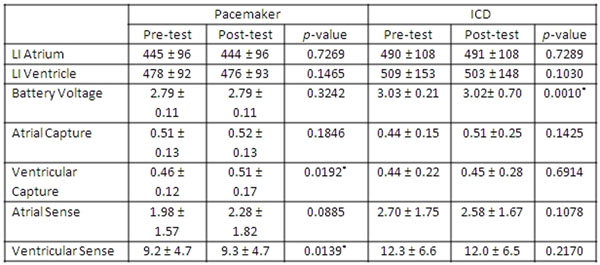
Device parameters for PM and ICD measures before and immediately after CMR.

**Figure 2:**
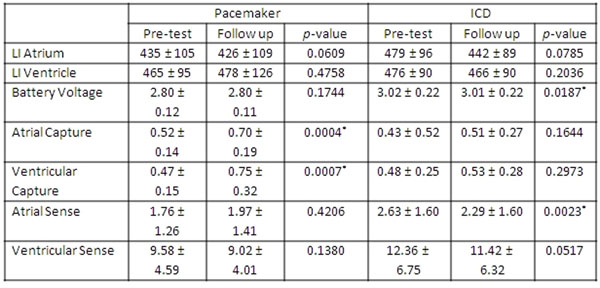
Device parameters for PM and ICD measures at follow up compared with the pre-test values.

## Conclusions

Patients with PM or ICD may safely undergo CMR using a pre-designed protocol without any clinically meaningful change in device function.

## Funding

None.

